# Racial Disparities in Asthma Hospitalizations Following Implementation of the Smoke-Free Air Law, Michigan, 2002–2012

**DOI:** 10.5888/pcd12.150144

**Published:** 2015-11-19

**Authors:** Michelle E. Marchese, Farid Shamo, Corinne E. Miller, Robert L. Wahl, Yun Li

**Affiliations:** Author Affiliations: Michelle E. Marchese, Corinne E. Miller, Robert L. Wahl, Michigan Department of Community Health, Lansing, Michigan; Yun Li, University of Michigan School of Public Health, Ann Arbor, Michigan.

## Abstract

**Introduction:**

Exposure to secondhand smoke has immediate adverse respiratory and cardiovascular effects. A growing body of literature examining health trends following the implementation of public smoking bans has demonstrated reductions in the rates of myocardial infarction and stroke, but there has been no extensive work examining asthma hospitalizations. The aim of this study was to determine the impact of the Michigan Smoke-Free Air Law (SFA law) on the rate of asthma hospitalizations among adults in Michigan and to determine any differential effects by race or sex.

**Methods:**

Data on adult asthma hospitalizations were obtained from the Michigan Inpatient Database (MIDB). Poisson regression was used to model relative risks for asthma hospitalization following the SFA law with adjustments for sex, race, age, insurance type, and month of year. Race-based and sex-based analyses were performed.

**Results:**

In the first year following implementation of the SFA law, adjusted adult asthma hospitalization rates decreased 8% (95% confidence interval [CI], 7%–10%; *P* < .001). While asthma hospitalization rates for both blacks and whites declined in the 12 months following implementation of the SFA law, blacks were 3% more likely to be hospitalized for asthma than whites (95% CI, 0%–7%; *P* = .04). The rate of decline in adult asthma hospitalizations did not differ by sex.

**Conclusion:**

The implementation of the SFA law was associated with a reduction in adult asthma hospitalization rates, with a greater decrease in hospitalization rates for whites compared with blacks. These results demonstrate that the SFA law is protecting the public’s health and saving health care costs.

## Introduction

The negative health consequences from exposure to secondhand smoke are well documented. In addition to causing lung cancer, heart disease, and stroke, secondhand smoke exposure has been associated with increased risk for other cancers, premature birth, infertility, and respiratory disease (1,2). To reduce the public’s exposure to secondhand smoke, 26 states have banned smoking in bars, restaurants, and worksites, and another 10 states have implemented partial smoking bans in these places (3).

Dozens of studies have examined whether public smoking bans have changed the number of myocardial infarction and stroke events, but less research has reported on asthma events (4,5). Tobacco smoke is a known asthma trigger, so it is reasonable to expect a decrease in asthma hospitalizations when exposure to secondhand smoke is restricted. The first study to investigate the effects of a smoking ban on asthma examined the smoking ban in Lexington-Fayette County, Kentucky, using emergency department data and reported a 24% decline in adult emergency department visits after the ban (6). Other studies examining asthma rates following state, province, and country-wide smoking bans have reported 0% to 22% reductions in asthma hospitalization rates (5,7–10). To date, there are no comprehensive analyses by race and sex of asthma hospitalization outcomes following a smoking ban. In 2013 in Michigan, 11.5% of the adult population self-reported currently having asthma, the third highest asthma prevalence in the country (11). Although the prevalence of asthma in Michigan black and white adult populations is similar (10.7% vs 9.5%, respectively), black adults are more than 4 times as likely to be hospitalized for asthma (12). Michigan women are hospitalized slightly more often than men (19.3 per 10,000 vs 7.7 per 10,000), but women also have a higher asthma prevalence (12.6% vs 7.0%) (12). Asthma hospitalizations have been increasing in the adult Michigan population since 2000 (12).

The Michigan Smoke-Free Air Law (SFA law), implemented May 1, 2010, banned smoking in Michigan bars, restaurants, hotels and motels, bowling alleys, bingo halls, nonfood work places and other indoor public areas. The objective of this observational time series study was to examine the rate of adult asthma hospitalizations in Michigan before and after implementation of the SFA law and to determine any differences in adult asthma hospitalization rates by race or sex.

## Methods

Hospital discharge data were obtained from the Michigan Inpatient Database (MIDB) from January 2002 through December 2012. The MIDB is purchased by the Michigan Department of Community Health (MDCH) from the Michigan Health and Hospital Association (MHA). The MHA compiles the data from more than 95% of the hospitals in Michigan. Data available from these medical records include the patient’s admission date, primary discharge diagnosis, age, sex, race, and insurance type. The Division for Vital Records and Health Statistics (DVRHS) at MDCH reviews and edits the data in the MIDB. Age, sex, and race are verified with MDCH Vital Records, the Annual Survey of Health Facilities conducted by MDCH, National Editing Procedure from the National Center for Health Statistics, hospital medical records personnel, and census data from the US Bureau of the Census. About 25% of all MIDB records are missing race data: DVRHS can match almost half of these missing cases (12% of total cases) to known race data from the Michigan Birth Registry, the Michigan Death Registry, and from a patient’s previous hospital visit where race information was recorded. Race is imputed for the remaining 13% of unknown cases according to the known proportion of hospitalizations by zip code, race, age, and sex. Tobacco use data were not available from the MIDB. This study was declared exempt from full review by the MDCH Institutional Review Board for the Protection of Human Research Subjects.

Asthma hospitalizations were determined by the primary discharge diagnosis for asthma (*International Classification of Diseases, Ninth Revision*, codes 493.xx). To study adult asthma while minimizing the potential confounder of COPD, we limited the study to those aged 20 to 64 years.

We used Poisson regression to model monthly asthma hospitalization rates during our 11-year study period. Because we were analyzing counts in a statewide population, we specified Poisson distribution for the model. Population offset was determined by the annually published MDCH DVRHS intercensal estimates for age, race, and sex (13). These estimates are bridged for race by using the National Center for Health Statistics methodology (14). Because we adjusted for age in our model, we based the regression on unadjusted asthma hospitalization rates. To study the effect of the law, we employed an indicator variable set as 0 for preban (months from January 2002 to before May 2010) and 1 for postban (May 2010 through December 2012). To account for the increase in Michigan adult asthma hospitalizations since 2000, we included a time variable (1 to 132 months) and an interaction term between the ban and time (ban × time) in our model. We ran a second model where we added the ban term as a separate term (ban + ban × time), but the ban term was not significant. Therefore, we present the first model in this article (ban × time, with no separate ban term); this model is more parsimonious.

Analyses were adjusted for age, sex, race, insurance type, and month of year (1 to 12). We adjusted for month of year to model the seasonal pattern of asthma hospitalizations (15). Age adjustments were determined by the following intercensal population estimate age groups (20–24, 25–29, 30–34, 35–39, 40–44, 45–49, 50–54, 55–59, and 60–64). Separate analyses stratified the population by race (black, white), or sex (male, female) and adjusted for age, insurance type, and month of year (1 to 12). In the analysis that stratified the population by race, we also adjusted for sex, and in the analysis that stratified by sex, we also adjusted for race.

We used Healthcare Cost and Utilization Project data to determine the average charge for an asthma hospitalization in Michigan (16). This charge was multiplied by the calculated difference between actual hospitalizations through 2012 and our projected number of asthma hospitalizations had the SFA law not been enacted. We present this value as the total amount of health care costs saved after the implementation of the SFA law.

Statistics were computed using SAS version 9.2 (SAS Institute Inc).

## Results

From January 2002 through December 2012, there were 88,438 hospitalizations of patients aged 20 to 64 years in the MIDB with a primary discharge diagnosis of asthma (Table 1). Women, blacks, and patients insured by Medicaid or Medicare were hospitalized at higher rates than men, whites, and privately insured patients (Table 1). Race data were missing from 0.2% of events that were analyzed.

**Table 1:** Characteristics of People Hospitalized for Asthma (N = 88,438), Michigan, January 2002–December 2012^a^

Characteristic	No. (%) of Hospitalizations	No. of Hospitalizations per 10,000 Population
**Sex**		
Male	24,913 (28.2)	7.67
Female	63,525 (71.8)	19.28
**Race**		
Black	38,326 (43.3)	41.26
White	48,396 (54.7)	8.98
Other	1,564 (1.8)	6.88
Missing	152 (0.2)	NA
**Age, y**		
20–24	4,482 (5.1)	5.96
25–29	5,435 (6.1)	8.12
30–34	6,398 (7.2)	9.36
35–39	8,789 (9.9)	11.97
40–44	11,800 (13.3)	14.65
45–49	14,221 (16.1)	16.84
50–54	14,524 (16.4)	18.04
55–59	12,441 (14.1)	17.85
60–64	10,348 (11.7)	18.68
**Insurance^b^**		
Private	38,024 (43.0)	8.53
Medicaid	22,061 (24.9)	29.18
Medicare	18,912 (21.4)	49.76
Other	9,392 (10.6)	NA
Missing	49 (0.1)	NA

Abbreviation: NA, not applicable.

a Michigan Health and Hospital Association (MHA). The MHA compiles the data from more than 95% of the hospitals in Michigan.

b Insurance data were from the US Bureau of the Census, 2012 American Community Survey (17) .

After adjusting for age, sex, race, insurance type, and month of year, there was an 8% reduction in the population-wide rate of asthma hospitalizations in the 12 months after the implementation of the SFA law (RR, 0.92; 95% confidence interval [CI], 0.90–0.93; *P* < .001) (Table 2). In the first 12 months of the ban, black hospitalization rates decreased 7% (RR, 0.93; 95% CI, 0.91–0.95; *P* < .001) and white hospitalization rates decreased 10% (RR, 0.90; 95% CI, 0.88–0.92; *P* < .001) (Table 2). The rate of hospitalizations for men and women were significantly reduced, but no differences in rates were found between the sexes (data not shown).

**Table 2:** Full Multivariable Poisson Regression Models for Relative Risks for Asthma Hospitalization for the First 12 Months Following Implementation of the Smoke Free Air Law (May 2010–April 2011), by Race, Michigan^a^

Category	All		Black		White	
	RR (95% CI)	*P*	RR (95% CI)	*P*	RR (95% CI)	*P*
**Sex**						
Male	1 [Reference]					
Female	2.39 (2.35–2.42)	<.001	1.98 (1.94–2.02)	<.001	2.77 (2.71–2.82)	<.001
**Age group, y**						
20–24	1 [Reference]					
25–29	1.36 (1.30−1.41)	<.001	1.30 (1.23–1.38)	<.001	1.40 (1.33–1.48)	<.001
30–34	1.57 (1.51–1.63)	<.001	1.47 (1.39–1.55)	<.001	1.66 (1.58–1.75)	<.001
35–39	2.04 (1.97–2.11)	<.001	2.09 (1.99–2.21)	<.001	2.01 (1.91–2.11)	<.001
40–44	2.59 (2.50–2.68)	<.001	2.84 (2.70–2.99)	<.001	2.42 (2.30–2.54)	<.001
45–49	3.01 (2.91–3.11)	<.001	3.47 (3.30–3.64)	<.001	2.72 (2.60–2.85)	<.001
50–54	3.23 (3.12–3.34)	<.001	3.56 (3.39–3.74)	<.001	3.04 (2.90–3.19)	<.001
55–59	3.21 (3.10–3.32)	<.001	3.26 (3.10–3.43)	<.001	3.21 (3.06–3.37)	<.001
60–64	3.40 (3.29–3.53)	<.001	3.24 (3.07–3.42)	<.001	3.57 (3.40–3.75)	<.001
**Race**						
White	1 [Reference]					
Black	4.72 (4.66−4.79)	<.001	NA			
Other	0.05 (0.05–0.05)	<.001				
**Insurance**						
Private	1 [Reference]					
Medicaid	0.50 (0.49–0.51)	<.001	0.57 (0.55–0.59)	<.001	0.45 (0.44–0.46)	<.001
Medicare	0.58 (0.57–0.59)	<.001	0.82 (0.80–0.84)	<.001	0.43 (0.42–0.44)	<.001
Self-Pay	0.18 (0.17–0.18)	<.001	0.24 (0.23–0.25)	<.001	0.14 (0.14–0.15)	<.001
Other	0.07 (0.07–0.07)	<.001	0.11 (0.10–0.11)	<.001	0.04 (0.04–0.05)	<.001
Missing	0.00 (0.00–0.00)	<.001	0.00 (0.00–0.00)	<.001	0.00 (0.00–0.00)	<.001
**Month of hospitalization**						
January	1 [Reference]					
February	1.06 (1.02–1.09)	.001	0.99 (0.95–1.04)	.82	1.10 (1.06–1.15)	<.001
March	1.08 (1.05–1.12)	<.001	1.01 (0.96–1.06)	.82	1.14 (1.10–1.19)	<.001
April	0.96 (0.93–0.99)	.02	0.96 (0.91–1.00)	.08	0.97 (0.93–1.01)	.19
May	0.96 (0.93–1.00)	<.001	1.00 (0.96–1.05)	.86	0.94 (0.90–0.98)	.003
June	0.83 (0.81–0.86)	<.001	0.91 (0.87–0.96)	<.001	0.77 (0.74–0.81)	<.001
July	0.71 (0.69–0.74)	<.001	0.79 (0.75–0.83)	<.001	0.65 (0.62–0.68)	<.001
August	0.73 (0.70–0.75)	<.001	0.80 (0.76–0.85)	<.001	0.66 (0.63–0.70)	<.001
September	1.02 (0.99–1.06)	.16	1.06 (1.01–1.11)	.01	0.99 (0.95–1.03)	.67
October	1.11 (1.08–1.15)	<.001	1.14 (1.09–1.19)	<.001	1.10 (1.05–1.14)	<.001
November	0.92 (0.89–0.95)	<.001	0.92 (0.87–0.96)	.001	0.93 (0.89–0.97)	.001
December	0.93 (0.90–0.96)	<.001	0.92 (0.88–0.97)	.002	0.93 (0.89–0.97)	.001
**Time**	1.00 (1.00–1.00)	<.001	1.00 (1.00–1.00)	<.001	1.00 (1.00–1.00)	<.001
**Time × ban**	**0.92 (0.90–0.93)**	**<.001**	**0.93 (0.91–0.95)**	**<.001**	**0.90 (0.88–0.92)**	**<.001**

Abbreviations: CI, confidence interval; NA, not applicable; RR, relative risk.

a The models were adjusted for age, sex, race (in the All category only), insurance type, and month of year.

**Figure 1:**
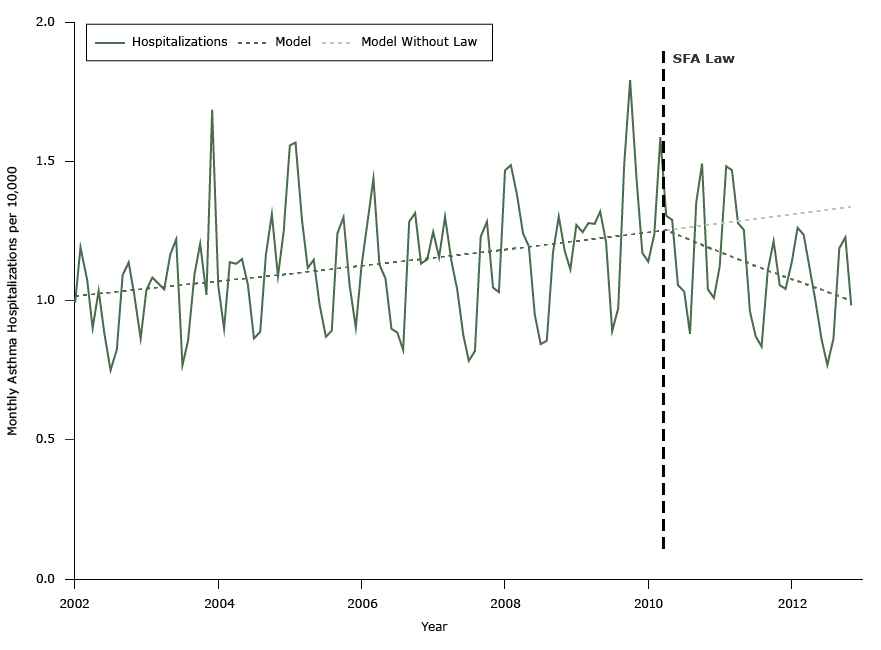
Actual and unadjusted modeled rate of asthma hospitalizations per 10,000 population per month among adults aged 20 to 64 years before and after implementation of the Smoke Free Air law (SFA law) (May 2010) and projected hospitalizations without the law, Michigan, January 2002–December 2012.

Before implementation of the SFA law, the population-adjusted monthly hospitalization rate increased with time; however, in the 32 months following the SFA law, the asthma hospitalization rate declined significantly (Figure 1).

**Figure 2:**
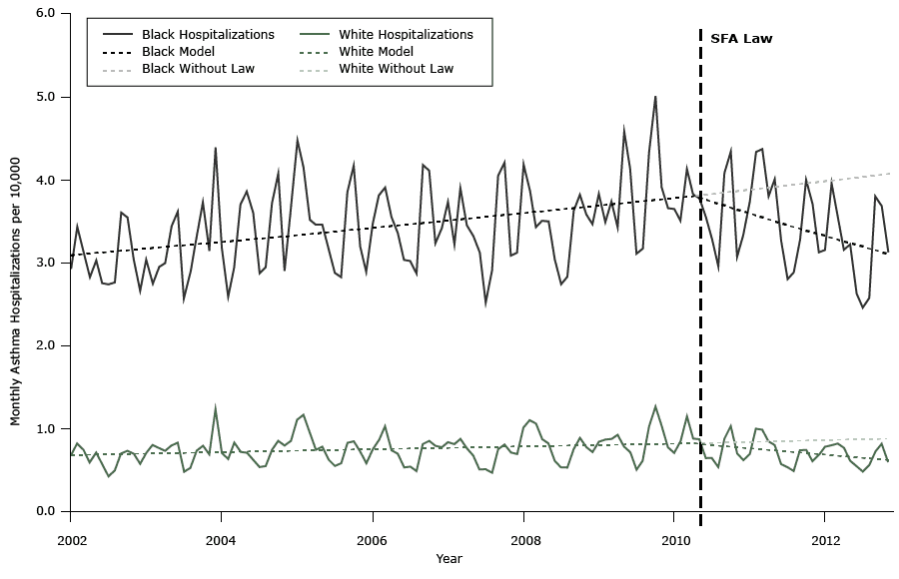
Actual and unadjusted modeled rate of asthma hospitalizations, by race, per 10,000 population per month among adults aged 20 to 64 years before and after implementation of the Smoke Free Air law (SFA law) (May 2010) and projected hospitalizations without the law, Michigan, January 2002–December 2012.

The black population had more than 4 times as many asthma hospitalizations per 10,000 persons as the white population (Figure 2). Following implementation of the SFA law, blacks and whites both had a reduced rate of asthma hospitalization; however, blacks were 3% more likely to be hospitalized than whites (95% CI, 0%–7%; *P* = .04). While blacks had a greater reduction in the absolute numbers of asthma hospitalizations, whites had a greater decrease in relative risk for hospitalization, with the annual decrease in hospitalization rates at 10% in whites versus 7% in blacks.

We found the average charge for hospitalizations in Michigan citing asthma as the primary diagnosis to be $15,047 per one hospital stay in 2011 dollars. We found a reduction of 3,230 adult asthma hospitalizations in the 32 months following implementation of the SFA law, which saved approximately $48.6 million in health care costs.

## Discussion

In the 32 months following implementation of the SFA law, hospitalization rates for asthma among adults in Michigan decreased by 21% after adjusting for age, sex, race, insurance type, and month of year. Although the reduction in the absolute number of hospitalizations was greater among blacks than among whites, the reduction in the relative risk among whites was greater. The rate of decline in asthma hospitalizations was the same in men and women.

Several studies found that the implementation of an SFA law is associated with a reduction of asthma-related medical care (6–9,18–21). However, this study is the first to use statewide data on asthma hospitalizations and to show a racial disparity in health benefits resulting from the SFA law. Because blacks have a higher smoking prevalence than whites, we expected to find a greater decrease in asthma hospitalizations for blacks after the ban (22). Before the statewide ban on public smoking, 25 Michigan counties and 2 major cities (Detroit and Grand Rapids) had smoke-free ordinances. Most blacks in Michigan reside in the urban areas that were affected by these smoke-free ordinances. Therefore, it is possible that the reductions in asthma hospitalizations that we are reporting were dampened by these previous ordinances, and this dampening disproportionately affected the black population. SFA laws have been shown to encourage smokers to reduce smoking or quit smoking altogether (23,24). If race is a marker for economic status, whites may have more resources to help them quit smoking. Likewise, more middle-class and upper-class people will patronize bars and restaurants and benefit from the reduction in secondhand smoke exposure.

The 8% decrease in hospitalizations that we have reported is within the range of other asthma studies following an SFA law (4). People with asthma who are hospitalized have poorly controlled asthma and represent the most severe cases. Our next steps at MDCH are to study the change in emergency department visits following implementation of the SFA law. Most emergency department visits for asthma do not result in hospitalization; emergency department visits for asthma are greater in number than hospitalizations for asthma. Also, the number of visits for asthma is more likely to change with changes in the environment (25).

Our study is strengthened by the large data set available to us. We examined statewide hospitalization data for 100 months preceding the SFA law and 32 months following the ban. Because this is a multiyear study, we were able to adjust for seasonality, which is a known cofactor in the assessment of the effects of environmental exposures on asthma hospitalization rates (15).

This research has limitations. As an observational study, we can determine only an association between implementation of the SFA law and the reduction in hospitalizations, we cannot determine causality. The Michigan Behavioral Risk Factor Survey (MiBRFS) can be used to assess temporal changes in tobacco use patterns; however, because of changes in MiBRFS methodology in 2011, we cannot accurately evaluate these temporal changes following implementation of the SFA law. A portion of the race data that we used (13%) was imputed. Because we did not have access to the data before the race imputation, we were unable to perform a sensitivity analysis on the data set to determine that racial disparity in hospitalization events was consistent without the imputed hospitalizations. Additionally, MIDB data do not include patient smoking status, so we are unable to differentiate whether changes in asthma hospitalizations reflect changes in smoking behavior or secondhand smoke exposure.

This study contributes to a growing body of literature associating smoke-free legislation with improved public health and decreased health care costs. We were surprised to find such a significant health disparity by race following the SFA law. More work is necessary to understand the causes for these disparate hospitalization rates to promote the health benefits to all Michigan populations.
